# Deltamethrin Resistance Mechanisms in *Aedes aegypti* Populations from Three French Overseas Territories Worldwide

**DOI:** 10.1371/journal.pntd.0004226

**Published:** 2015-11-20

**Authors:** Isabelle Dusfour, Pilar Zorrilla, Amandine Guidez, Jean Issaly, Romain Girod, Laurent Guillaumot, Carlos Robello, Clare Strode

**Affiliations:** 1 Institut Pasteur de la Guyane, Unité d’Entomologie Médicale, Cayenne, French Guiana, France; 2 Unidad de Biología Molecular, Institut Pasteur de Montevideo and Dept. of Biochemistry, School of Medicina, Montevideo, Uruguay; 3 Institut Pasteur de Nouvelle Calédonie, Unité de Recherche et d’Expertise en Entomologie Médicale, Noumea, New Caledonia; 4 Biology Department, Edge Hill University, Ormskirk, Lancashire, United Kingdom; Centers for Disease Control and Prevention, UNITED STATES

## Abstract

**Background:**

*Aedes aegypti* is a cosmopolite mosquito, vector of arboviruses. The worldwide studies of its insecticide resistance have demonstrated a strong loss of susceptibility to pyrethroids, the major class of insecticide used for vector control. French overseas territories such as French Guiana (South America), Guadeloupe islands (Lesser Antilles) as well as New Caledonia (Pacific Ocean), have encountered such resistance.

**Methodology/Principal Findings:**

We initiated a research program on the pyrethroid resistance in French Guiana, Guadeloupe and New Caledonia. *Aedes aegypti* populations were tested for their deltamethrin resistance level then screened by an improved microarray developed to specifically study metabolic resistance mechanisms. Cytochrome P450 genes were implicated in conferring resistance. *CYP6BB2*, *CYP6M11*, *CYP6N12*, *CYP9J9*, *CYP9J10* and *CCE3* genes were upregulated in the resistant populations and were common to other populations at a regional scale. The implication of these genes in resistance phenomenon is therefore strongly suggested. Other genes from detoxification pathways were also differentially regulated. Screening for target site mutations on the voltage-gated sodium channel gene demonstrated the presence of I1016 and C1534.

**Conclusion /significance:**

This study highlighted the presence of a common set of differentially up-regulated detoxifying genes, mainly cytochrome P450 genes in all three populations. GUA and GUY populations shared a higher number of those genes compared to CAL. Two *kdr* mutations well known to be associated to pyrethroid resistance were also detected in those two populations but not in CAL. Different selective pressures and genetic backgrounds can explain such differences. These results are also compared with those obtained from other parts of the world and are discussed in the context of integrative research on vector competence.

## Author Summary

*Aedes aegypti* is vector of Dengue, Chikungunya and Zika viruses, all causing emerging or re-emerging diseases worldwide. Fighting these diseases relies on the control of the vector. Therefore, insecticides have been extensively used worldwide, resulting in the development of insecticide resistance. In the French overseas territories, resistance to pyrethroids has been monitored for many years with high levels in the South American French territories. We then investigated the mechanisms underlying this resistance in populations from French Guiana, Guadeloupe and New Caledonia. Transcription levels of detoxification genes were measured and alongside screening for target site mutations. Upregulation of cytochrome P450 genes and carboxylesterases were observed in all three populations. Mutations related to pyrethroid resistance in position 1016 and 1534 of the voltage-gated sodium channel gene were also observed. French Guiana and Guadeloupe populations presented a closer profile of resistance mechanisms whereas the New Caledonia population had a more restricted profile. Such differences can be explained by different vector control practices, regional insecticide uses and genetic backgrounds. These results are also compared with others obtained from other parts of the world and are discussed with the perspective of integrative research on vector competence.

## Introduction

*Aedes (Stegomyia) aegypti* (Linnaeus, 1762) is a mosquito species of high medical importance due to its widespread distribution and ability to transmit a variety of arboviruses. For decades its control has involved mechanical elimination of breeding sites as well as larvicidal applications and adulticide spatial spraying operations. However, the efficacy of these insecticide treatments has been reduced due to the development of resistance in this species. French overseas territories such as French Guiana, Martinique and Guadeloupe (French Territories in the Americas, FTAs), and New Caledonia (West Pacific) have all experienced insecticide resistance in *Ae*. *aegypti* populations [1–4] over the course of vector control programmatic changes. Since the 1940s, all the territories that once used organochlorine (OC), organosphosphate (OP), pyrethroids (PY) and bioinsecticides insecticides successfully have observed the development of vector resistance to the majority of them with the exception of bio-insecticides. Since the prohibition of the sale and use of many biocide products by the European Community (EC), the FTAs are facing a dilemma in their vector control strategies. Despite the fact that pyrethroids have the highest level of resistance in *Ae*. *aegypti*, they are the only insecticide family authorised for mosquito control.

Vector control strategies depend on the decisions of local authorities, which differ from one territory to another. The French Guianan program of surveillance and management of dengue cases conducts *Ae*. *aegypti* density reduction throughout the year which is intensified during outbreaks. Vector control activities include both indoor and outdoor spatial spraying of deltamethrin (PY) against adults and the removal of breeding sites or their treatment with *Bacillus thuringiensis* var. *israelensis* (Bti) based larvicides. Deltamethrin is also used routinely for pest mosquito management. In contrast, the territory of Guadeloupe limits the use of insecticides to only during dengue and other arbovirosis epidemics and focuses on larval elimination during non-epidemic periods. In New Caledonia, where EC regulations do not apply, the local government conducts regular monitoring of insecticide resistance that has led to a switch from deltamethrin to malathion (OP) in zones where PY resistance was detected in 2003. Adulticides are sprayed in a 100 meter-radius around dengue, Chikungunya or Zika fever cases, which is coupled with mosquito source reduction and community awareness raising campaigns.

Pyrethroid resistance is explained by two main mechanisms. One is the alteration of the membrane voltage-gated sodium channel inducing insensitivity to the insecticide and absence of knock-down effect (*kdr*). This mechanism confers cross-resistance to PYs and DDT [5]. The second recognized mechanism is an overproduction of detoxifying enzymes, also called metabolic resistance. These enzymes are naturally involved in the degradation or transformation of toxic compounds into non-toxic products before elimination or sequestration into the insect body. Three large enzyme families, the cytochrome P450 monooxygenases (P450s), Glutathione S-transferases (GSTs) and carboxy/cholinesterases (CCEs) have been implicated in the metabolism of insecticides in insects. Throughout the species’ range, *Ae*. *aegypti* populations have shown strong resistance to pyrethroid, carbamate and organophosphate insecticides correlated with elevated activities of detoxifying enzymes and *kdr* mutations [6–11]. Monitoring insecticide resistance in mosquito populations and understanding the mechanisms involved is a prerequisite to efficient implementation of vector control strategies.

Transcriptomic tools such as the “*Aedes* detox chip” [12] are useful to identify detoxification gene regulation in insecticide resistant populations. However, while some of these genes are related to insecticide resistance, others are a response to environmental and evolutionary factors. The study of several mosquito populations could identify a common pattern of genes associated with pyrethroid resistance and eliminate those associated with environmental factors.

Therefore, the objective of this work is to obtain and compare data on pyrethroid resistance and the underlying resistance mechanisms in three *Ae*. *aegypti* populations collected in geographically distinct French overseas territories.

## Materials and Methods

### Ethics statement

Mosquito blood feeding is done on mice. We hold experimental authorization under the agreement number B973-02-01 delivered by "la préfecture de Guyane" and renewed on June 6th, 2015. Experimental project was re-approved by the ethical committee CETEA Institut Pasteur (n° 89), report number 2015–0010 issued on May 18th, 2015.

### Mosquito strains, collection and rearing conditions

*Aedes aegypti* mosquitoes were collected at the larval stage in Cayenne (French Guiana, GUY), Baie Mahault (Guadeloupe, GUA) and Noumea (New Caledonia, CAL) (S1 Fig). Discarded freezers, tyres and plant pots were the targeted breeding sites. Adults of the F0 generation were reared at each partner facility and allowed to mate. Females were provided a blood meal from mice and eggs of the F1 generation were produced. These eggs were shipped to the Institute Pasteur in French Guiana. Adults of the F2 generation were then obtained by rearing immature stages from the three populations concurrently under insectary conditions in French Guiana (28 ± 1°C, 70 ± 10% RH, 12:12h photoperiod). New Orleans (NO) susceptible strain was reared under the same conditions at the same time and used as a reference in all experiments.

### Assessment of insecticide resistance status

Insecticide resistance bioassays were carried out once per population through tarsal contact tests using filter papers impregnated with technical grade deltamethrin (CAS # 52918-63-5) (Sigma-Aldrich, St Louis, MO, USA) at the diagnostic dose of 0.06% as published in Jirakanjanakit et al. [13]. Filter papers were impregnated following the WHO protocol [14] using acetone solutions of insecticide and silicone oil as the carrier. Impregnation was conducted by dropping 2 mL of a solution containing technical grade chemical dissolved in acetone and silicone oil evenly onto each paper (12 x 15 cm). Concentrations were expressed in w/v percentage of the active ingredient in silicone oil. The papers were dried for 24 hours before the test. All the mosquito populations were exposed to the same batch of impregnated papers during one week. Following the WHO tube test protocol [15,16], four batches of 25 non-blood fed females (2–5 days old) were introduced into holding tubes for one hour then transferred into the exposure tube and placed vertically for one hour. Knocked-down and dead mosquitoes were recorded after this time (1h KD) before being transferred back to the holding tubes. A mosquito was recorded as knocked-down if it was lying on its back or side and was unable to instigate flight after a gentle tap. Mortality was recorded 24 hours after exposure (24h M). Controls, made of only acetone and silicone oil delivered to the filter paper, were performed as mentioned above with a total of two batches of 25 non-blood fed females (2–5 days old) per replicate. All replicates were conducted at 27 ± 2°C and a relative humidity of 60 ± 10%. A ten percent sugar solution on soaked cotton balls was provided to the females during the 24 hours observation period.

### Mosquito processing prior to *kdr* genotyping and microarrays experiments

Three day old females were exposed to 0.06% deltamethrin impregnated filter papers in the WHO tubes as described above. Up to 100 specimens surviving after 48 hours of observation were anesthetized on ice, dropped into RNAlater (Qiagen, Redwood city, CA, USA) and preserved following manufacturer instructions. Samples were shipped by express mail to the Institute Pasteur de Montevideo for microarray experiments. Other specimens were separated into surviving (resistant), dead (susceptible) and non-exposed (control) mosquitoes and stored dry prior to *kdr* genotyping.

### Genotyping of the mutations on the sodium channel gene

DNAzol (Life Technologies, Gaithersburg, MD, USA) was used for total DNA isolation of resistant and susceptible individuals prior to sequencing the fragment encompassing exon 20 and exon 21 of the sodium channel voltage dependent gene (Nav). Primers used to amplify this fragment were AaNaA 5’-ACAATGTGGATCGCTTCCC-3’and AaNaB 5’-TGGACAAAAGCAAGGCTAAG-3’(8). The primers amplify a fragment of approximately 472 bp which includes an intron of approximately 28 bp. For PCR reactions, 100 ng of DNA were used as a template, and samples were incubated for 3 min at 95°C followed by 30 amplification cycles of 30 sec at 94°C, 30 sec at 60°C and 1 min at 72°C, with a final extension of 5 min at 72°C. PCR products were loaded on a 4% gel made of low-melting temperature agarose (NuSieve, Lonza, Rockland, ME, USA) and migrated to separate fragments with introns of two sizes. Fragments were isolated, purified, directly sequenced, and sequences were aligned by using ClustalW [17].

Based on these sequences, we designed primers and probes designed to develop an Allelic discrimination assay using the Taqman technology (Applied Biosystems, Foster City, CA, USA). The allelic discrimination assay was composed of two standard oligonucleotides V1016I SNP-F-GCT-AAC-CGA-CAA-ATT-GTT-TCC-C and V1016I SNP-R- CAG-CGA-GGA-TGA-ACC-GAA-AT. Each probe consists of a 5’ reporter dye, a 3’ non fluorescence quencher and a minor groove binder at the 3’end. The probe V1016-PV- CAC-AGG-TAC-TTA-ACC-TTT-T was labeled with 6-Vic dye fluorescence at the 5’ end for the detection of the wild-type allele whereas the probe I1016-PF-CAC-AGA-TAC-TTA-ACC-TTT-TC was labeled with FAM dye fluorescence at the 5’ end for the detection of the mutant allele. In addition, genotyping position 1534 on the domain III segment 6 was performed according to Yanola et al. [18]. The two standard oligonucleotides were F1534C SNP-F- GAT-GAT-GAC-ACC-GAT-GAA-CAG-ATC and F1534C SNP-R- CGA-GAC-CAA-CAT-CTA-GTA-CCT. Each probe consists of a 5’ reporter dye, a 3’ non fluorescence quencher and a minor groove binder at the 3’end. The probe F1534-PV- AAC-GAC-CCG-AAG-ATG-A was labeled with 6-Vic dye fluorescence at the 5’ end for the detection of the wild-type allele whereas the probe C1534-PF-ACG-ACC-CGC-AGA-TGA was labeled with FAM dye fluorescence at the 5’ end for the detection of the mutant allele.

For the allelic discrimination assay, DNA from individual adult mosquitoes was extracted with the Purelink Genomic DNA extraction kit (Invitrogen, Carlsbad, CA, *USA*) according to the manufacturer's instructions, with the following modification: cell lysis was performed manually with a sterile piston after addition of PureLink Genomic Digestion Buffer. DNA was isolated and suspended in 100 μl of PureLink Genomic Elution Buffer (from the kit) and stored at -20°C. The TaqMan reaction contained 12.5 μL of 2X TaqMan Universal Master Mix II (Life technologies, Gaithersburg, MD, USA), 1.44 μM of each primer, 0.4 μM of each probe and 3 μL of genomic DNA (20 ng) made up to 25 μL with sterile water. The assay was performed under discrimination allele settings using an StepOnePlus Real-Time PCR System (Life technologies, Gaithersburg, MD, USA), under the following thermocycling conditions: 10 min at 95°C, 45 cycles of 95°C for 15 sec and 60°C for 1 min. Data were analyzed by the StepOne Software version 2.1.

Relationships between phenotypes and putative resistant genotypes were tested using a Fisher’s test for each population with the MASS package in R 3.2.1 (R Development Core Team, Vienna, Austria). Linkage disequilibrium was tested with GENEPOP v4.2 [19,20].

### Microarrays experiment

#### RNA extraction and purification

Total RNA was extracted from 3-day-old female mosquito samples using the RNeasy Mini Kit from Qiagen, according to manufacturer’s instructions. RNA quality and integrity was analyzed using the Agilent 2100 Bioanalyzer (Agilent Technologies). RNA Integrity Number (RIN) was obtained for each sample, and samples with acceptable RINs were employed for the microarray experiments. RNA was also quantified using a spectrophotometer (NanoDrop 1000 Thermo Scientific).

#### Experiment design and microarray hybridization

The array used was an updated version of LSTM *Ae*. *aegypti* detox chip (A.-MEXP-623). The ‘*Ae*. *aegypti* detox chip plus’ (A-MTAB-574, http://www.ebi.ac.uk/arrayexpress)(8x15K Agilent Technologies) housed two 60-mer unique probes with a 3’bias for 1089 genes associated with responses to insecticide and xenobiotics identified from previous studies using including all cytochrome P450s, GST and COEs [12]. It also contained one probe for 2435 transporter genes and a unique probes for 222 ‘miscellaneous’ genes (associated with immunity, heat shock proteins and housekeeping).

A one-color microarray experiment was performed. Three sample groups corresponding to the three populations investigated (GUY, GUA and CAL) were all compared against the susceptible population from New Orleans (NO, reference strain). We used four biological replicates for each sample. In total, 16 one-color hybridizations were performed in this study. Experimental data are available under the accession number E-MTAB-4022.

The cDNA synthesis, labeling, hybridization and subsequent washing steps were performed using Agilent’s One-Color Microarray Gene Expression Analysis (Low input Labelling), according to the manufacturer’s instructions. Labeled cRNAs were hybridized to “*Ae*. *aegypti* Detox Chip Plus” to compare transcription levels of *Ae*. *aegypti* detoxification genes of the different populations against the susceptible New Orleans strain.

Arrays were scanned by the Agilent microarrays scanner G2565BA, using the default settings for all parameters. Raw data were collected using the Agilent Feature Extraction Software (Agilent Technologies, version 10.7).

#### Quality and statistical analysis of the microarray data

The quality of each hybridization was analyzed with Agilent Feature Extraction software using the information provided by the statistics and the spikes (known mixtures of RNAs used as positive controls in Agilent platforms).

Statistical analyses were performed with the Expression Analysis Software GeneSpring GX 12 (Agilent Technologies). Arrays were normalized by applying LOWESS normalization. To directly compare arrays, a median baseline transformation was performed. In addition, probes were filtered according to the following criteria: positive and uniform, not saturated and above the background signal. Unpaired T-tests were performed to compare the differential expression of genes from each of the three populations of interest (GUA, GUY and CAL) separately, against the reference population (NO) using a Benjamini-Hochberg multiple testing correction, and a threshold of significance of 0.001.

#### Real time PCR data validation

Four mosquito samples, two used in microarrays and two new batches, were used to validate, by real-time PCR, some of the over-expressed and repressed genes in the GUA, GUY and CAL populations compared to the NO population.

For each sample, cDNA was synthesized by reverse transcription using the Superscript II μReverse Transcriptase (Invitrogen) with oligo dT primers and 400 ng of total RNA added as a template. Exon-junction primers were designed in order to avoid genomic DNA contamination, and on the basis of mosquito nucleotide sequences available from Vectorbase (http://aaegypti.vectorbase.org/). The primer sequences and expected product lengths are listed in S1 Table. Real-time PCR reactions were performed using 5 μL of KAPA SYBR FAST Universal 2X qPCR Master Mix (www.kapabiosystems.com), equal amounts of forward and reverse primers (10μM) and 1μL cDNA in a final volume of 10μL.

For calculation of expression levels, relative quantification method was used [21]. A calibration curve was constructed with a pool of all the cDNA samples for the PCR efficiency determination. Standard curves for each of the genes amplified were done using serial dilutions (1/10, 1/20, 1/50 and 1/100). The efficiency calculation was done from the slopes of the calibration curves, according to the equation (*E* = 10^[–1/slope]^) as described by Pfaffl [21]. The *RpS14* housekeeping gene (40S Ribosomal protein S14, AAEL005266) was used as an endogenous control. Each run of real-time PCR included gene expression measurement of *RpS14* and the target gene in the corresponding samples.

Standard amplification conditions were 30 sec at 95°C, and 40 cycles of 3 sec at 95°C and 60 sec at 60°C. After each PCR reaction, the corresponding dissociation curves were analyzed to ensure that the desired amplicon was being detected and to discard contaminating DNA or primer dimers. Samples were analyzed in duplicate in a 72-disk Rotor-Gene 6000 (Corbett Life Sciences).

## Results

### Insecticide resistance status

One hundred females were exposed to deltamethrin 0.06% for one hour and left in observation for 24 hours. The percentage of knocked-down mosquitoes after one hour exposure were 29%, 37% and 100% for GUY, GUA and CAL populations respectively, demonstrating the presence of knocked-down resistance in GUY and GUA but none in CAL. Twenty-four-hour mortalities were then, respectively, 26%, 42% and 96%. Thus, All three populations were resistant to deltamethrin according to the WHO criteria [15]. GUY and GUA populations can be characterized as highly resistant whereas CAL has a lower level of resistance.

### *kdr* genotyping

A total of 58 sequences from 32 individuals were analysed (Table 1). Haplotypes A and B were observed as characterized by Martins et al. [8] in each population including the NO reference strain. Only the GUA population has an evident unbalanced proportion with a frequency 0.92 for haplotypes A. In position 1016 of the sequence, valine, related to deltamethrin susceptibility, was the only amino-acid found in NO (N = 6), the susceptible reference strain, and in the CAL population (N = 5). Both valine and isoleucine, related to resistance, were found in GUY and GUA populations. We were unable to detect V1016G in our sequences. In addition, two mutation points described in *Ae*. *aegypti* were checked: S989P, described in mosquitoes in a deltamethrin selected strain from Thailand [22] and I1011M/V were described in Latin America [8,23]. In position 1011 of the amino-acid sequence, methionine was absent in the GUA population, found in GUY population at low frequency (freq_met_ = 0.11), and in equivalent proportion with isoleucine in the CAL population (freq_met_ = 0.40) (Table 1).

**Table 1:** characterization of sequences at the Na_V_ gene: number of individuals (N), frequencies of haplotype A and B, allelic frequencies at position 989, 1011 and 1016 of the amino-acid sequence. Ser: Serine, Pro: Proline, Ile: Isoleucine, Met: Methionine, Val: Valine.

		Haplotypes^1^		Position 989		Position 1011		Position 1016	
Population	N	A	B	Ser	Pro	Ile	Met	Val	Ile
**GUY**	**14**	0.6	0.4	1	0	0.89	0.11	0.43	0.47
**GUA**	**7**	0.92	0.08	1	0	1	0	0.07	0.43
**CAL**	**5**	0.50	0.50	1	0	0.60	0.40	1	0
**NO**	**6**	0.71	0.29	1	0	1	0	1	0
**Total**	**32**								

^**1**^ haplotype group A is 250 pb of length and B is 234 pb of length. Additionally, two synonymous transitions (a → g) in exon 20, positions 26 and 89, separate the sequences of the two groups (8)

Even if several studies [24,25] have related I1011M/V mutation to resistance to cypermethrin or permethrin, others did not demonstrate such association with pyrethroid and particularly deltamethrin resistance [8,23,26]. However, amino-acid changes in position 1016 and 1534 are of higher interest for resistance phenotype, considered alone or in combination [8,26,27]. These are the reasons why we focused on genotyping V1016I and F1534C loci. In addition to the 26 individuals fully genotyped by sequencing, 146 individuals were genotyped for the 1016 codon using Allelic Discrimination Assay, making a total of 172 individuals genotyped for this locus. A total of 161 individuals were also genotyped for the 1534 codon. Each individual was classified as resistant or susceptible according to if they were alive or dead 48 hours after their exposure to deltamethrin (see above).

The CAL population exhibited only the wild-type (homozygous susceptible) genotype at the two loci regardless of the phenotypic class. This observation is in accordance with the absence of *kd* resistance observed in the bioassays. The resistant allele, 1016I, was recorded in higher proportion in GUY and GUA resistant mosquitoes, 92% and 88% respectively, than in susceptible ones, 65% and 79%, respectively. The allele 1534C was fixed in the GUY population regardless of the phenotype, while in GUA it exhibited frequencies of 88% in resistant mosquitoes and 77% in susceptible ones. Considering the resistance allele recessive [23,25,28], the association between the phenotype and genotype was tested using Fisher’s test. Only V1016I in GUY population was significantly associated with resistant phenotypes (p = 0.0018).

**Fig 1:**
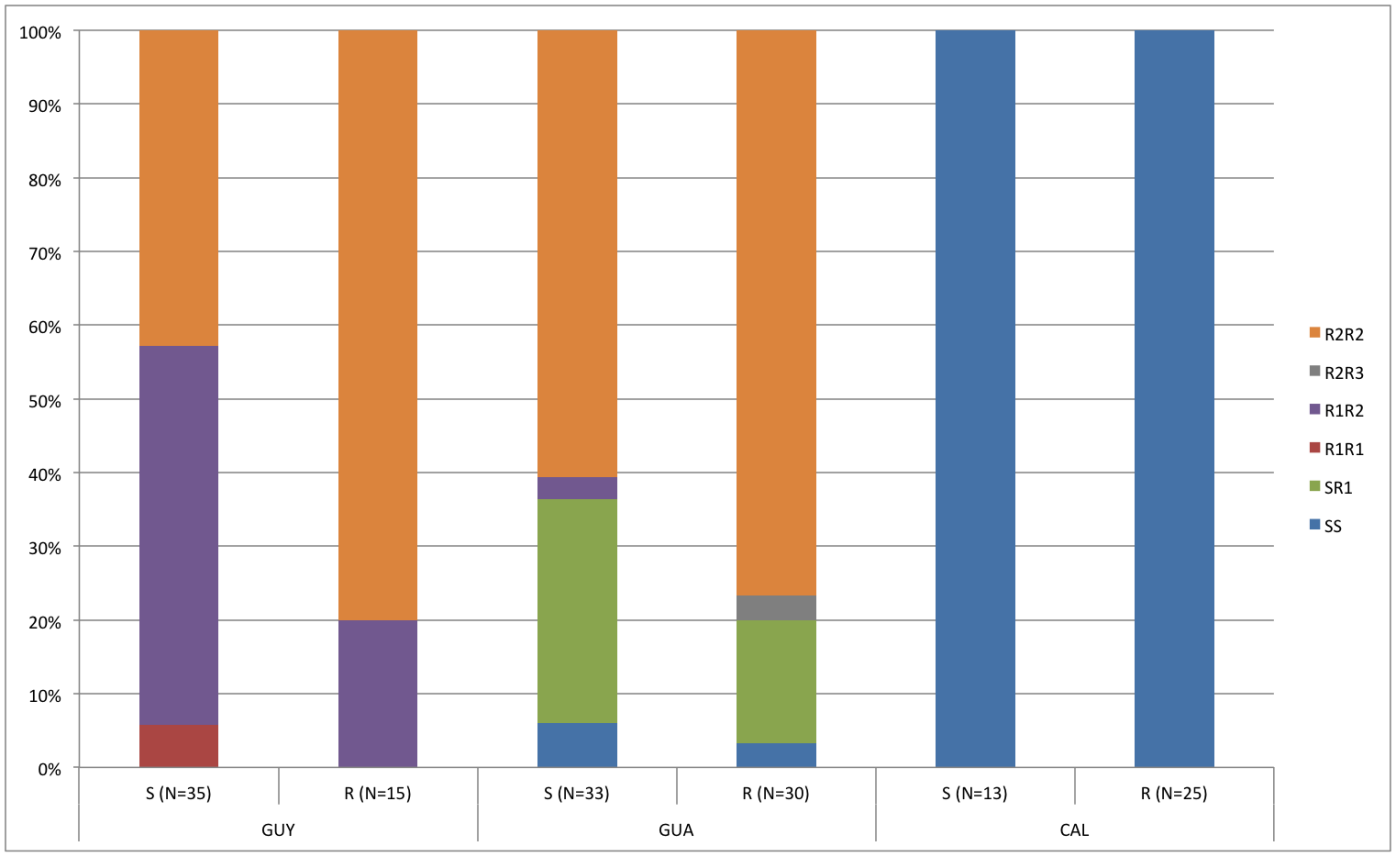
Cumulative histogram of genotype proportions per population and phenotype (S: susceptible, R: resistant). Number of individuals are also mentioned (N). Based on the description of Linss et al. [29], four alleles were obtained ‘1016V + 1534F’ called S, ‘1016V + 1534C’ (1534 *kdr*) called R1, ‘1016I + 1534 C’ referred to as R2 (1016 *kdr*+1534 *kdr*) and ‘1016I + 1534 F’ referred to as R3 (1016 *kdr*).

Significant linkage disequilibrium was observed between the two loci among all populations (p<0.001). Based of the description of Linss et al. [29], four alleles ‘1016V + 1534F’ called S, ‘1016V + 1534C’ (1534 *kdr*) called R1, ‘1016I + 1534 C’ referred to as R2 (1016 *kdr*+1534 *kdr*) and ‘1016I + 1534 F’ referred to as R3 (1016 *kdr*) were identified. Allele frequencies are shown in S2 Table. According to the alleles, the genotypes were named SS, SR1, R1R1, R1R2, R1R3 and R2R2. Genotype distributions in each population were different. As described above, the CAL population was genotypically fully susceptible. In contrast, the R2R2 genotype was observed in both GUY and GUA populations. However, R1R2 and R1R1 were observed in the GUY population while a majority of SR1 and SS were observed in the GUA population. R1R2 and R2R3 were scarcely represented in the GUA population, which was more diversified than the two others (Fig 1).

### Microarray overall analysis

Differences in gene expression of the GUY, GUA and CAL pyrethroid-resistant strains and the NO susceptible strain were assessed using a 15K ‘*Ae*. *aegypti* detox chip plus’ microarray platform containing 3746 unique genes. Using an arbitrary cut-off of fold change >2-fold in either direction and a t-test P-value of less than 0.001 after multiple testing correction, 63 (1.3%) genes were differentially transcribed between the GUY and NO strains (41 up regulated and 22 down regulated), 76 genes (1.5%) were differentially transcribed between the GUA and NO strains (48 up regulated and 28 down regulated) and 50 genes (1.5%) were differentially transcribed between the CAL and NO strains (39 up regulated and 11 down regulated). Of the 123 (2.51%) differentially regulated genes, 17 (2.5%) were in the three resistant populations: 12.2% were up regulated and 2.4% were down-regulated. In addition, 23 (18.7%) were common to GUY and GUA strains, 4 (3.3%) were common to GUY and CAL strains, 5 (4.1%) were common to GUA and CAL strains. Almost 16% of the differentially expressed genes are not annotated as ‘conserved hypothetical proteins’ or are specified as ‘metadata non available’ in Vectorbase (S3 Table). The expression data from these microarray experiments can be accessed at Vectorbase (E-MTAB-4022, http://www.vectorbase.org).

### Detoxification pathway genes

**Fig 2:**
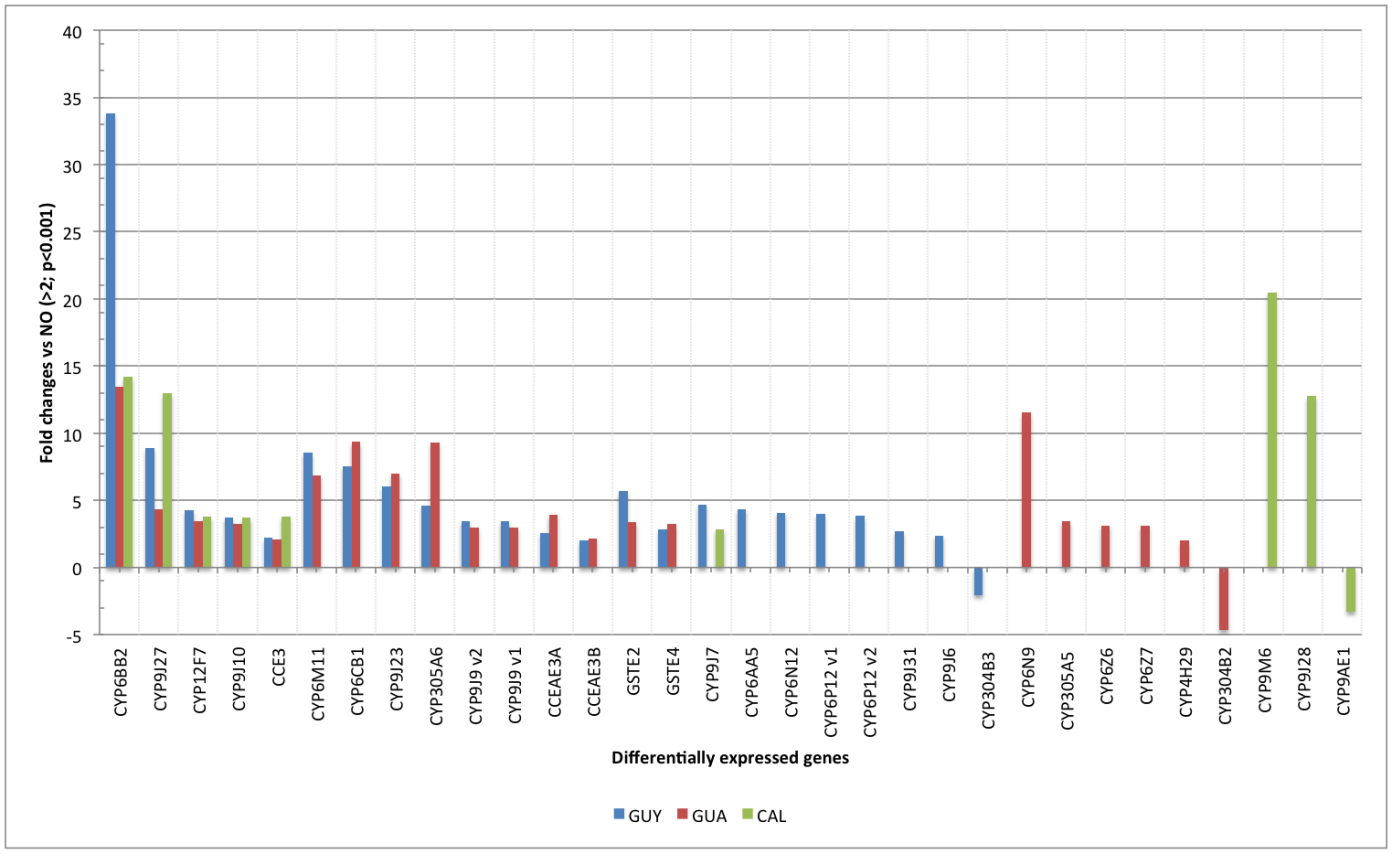
Fold changes of detoxification genes differentially expressed in pyrethroid resistant populations relative to the susceptible NO strain (Fold change>2; p<0.001). CYP: cytochrome P450 genes, CCE: carboxy/cholinesterase genes, GST: glutathione S-transferase genes.

**Fig 3:**
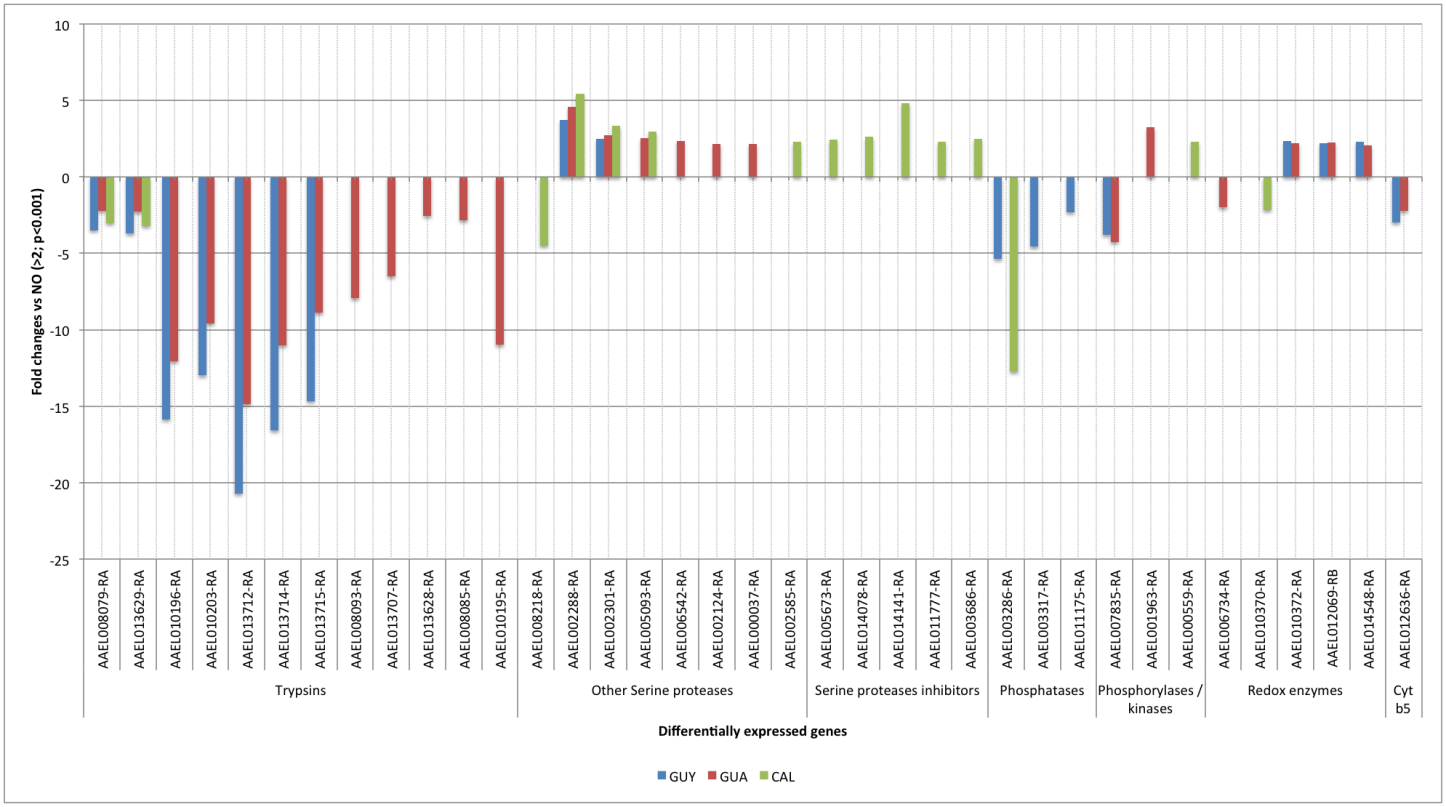
Fold changes of differentially expressed genes with a putative role in detoxification gene regulation or transformation of their products relative to the susceptible NO strain (Fold change>2; p<0.001).

A total of 27 genes from the cytochrome P450 family (CYP450), three from carboxylesterases (CCE) and 2 from Glutathione S-transferases (GST) were differentially regulated (Figs 2 and 3). One CYP450 gene was down regulated in each studied population. Nine genes from CYP9J, two from CYP6Z and two from CYP6M families were observed. Those families are important in detoxifying pyrethroids in mosquitoes. Among the genes common to the three studied populations, one was from CCE (*CCE3*) and four were CYP450 (*CYP12F7*, *CYP9J10* and *CYP9J27*, *CYP6BB2*). *CY6BB2* is noticeable with up-regulations respectively of 33.83, 13.47 and 14.19 fold *vs* the NO population in GUY, GUA and CAL populations. The CAL population is tolerant to deltamethrin, and apart from the five detoxification genes common to all populations, only four others are differentially expressed with only one common to GUY and none to the GUA population. *CYP9J28*, *CYP9J27* and *CYP6M9* were also up-regulated up to 12.78 to 20.48 fold compared to the NO strain. GUY and GUA populations were highly resistant to deltamethrin and had 10 additional differentially expressed genes in common (Fig 2).

Thirty-seven genes producing enzymes, which may play a role in the detoxification pathway as regulators or product transformations (redox enzymes) were differentially expressed in all three populations (Fig 3). Among them, 19 are from the serine protease family including 13 down-regulated genes and seven up-regulated. In the GUY population, seven are down-regulated and two up-regulated; in the GUA population, 12 are down-regulated and 6 up-regulated; in the CAL population, three are down-regulated and four up-regulated (Fig 3). Serine protease inhibitors are only up-regulated in the CAL population. Overall, 12 genes annotated as “trypsin” or “trypsin precursor” were down-regulated in the three populations. Two of those were common to the three studied populations; five were down-regulated from 9.58 to 20.71 fold in GUY and GUA populations; five were observed only in the GUA population. Two other serine proteases were over-expressed in the three populations.

Seven genes coding for phosphatases, phosphorylases, kinases and cytochrome b5 were up- or down-regulated (Fig 3). Those enzymes are known to play a role in regulating Cyp450 enzymes. Nine of those enzymes were down-regulated above 5-fold in both GUY and GUA populations whereas only two are differentially regulated in the CAL population. Finally, four of the five redox related genes code for aldehyde oxidase, glutathione peroxidase and thioredoxin peroxidase. They were mainly observed as up-regulated genes in GUY and GUA populations (Fig 3). Overall, detoxification profiles were more alike between GUY and GUA populations than with CAL populations.

## Discussion

The three *Ae*. *aegypti* populations studied are geographically isolated and are from areas with different vector control histories. Therefore, disparity between these areas was observed: high deltamethrin resistance levels were found in GUY and GUA compared to a lower level in CAL. While French Guiana and Guadeloupe have used organophosphates for a long time before switching to deltamethrin more recently, New Caledonia has managed the development of resistance by alternating and reducing the use of insecticides. In addition, *Ae*. *aegypti* resistance to deltamethrin in New Caledonia is only found in urban environments, which suggests a link with public health, domestic and gardening use of insecticides, as opposed to agricultural use of insecticides as the case in Guadeloupe. In French Guiana, agriculture is not so extensive which also suggests a limited impact on the development of *AE*. *AEGYPTI* resistance in that territory.

On account of the different strategies of insecticide use, resistance mechanism patterns are slightly different from one site to another but there is however a consistent underlying core. *Na*_*v*_ sequencing demonstrated the presence of haplotypes A and B [8] in each population with a lesser extent in Guadeloupe samples. In addition, S989P found in Asia associated with V1016G was not recorded in our study. I1011M wild type was found mainly in the FTA and in equivalent proportion in CAL. These two mutations were not further investigated due to scarce evidence of their impact on deltamethrin resistance. S989P was associated with V1016G mutation in Southeast Asia populations [30,31]. I1011M was associated with cypermethrin resistance in Brazil [25] and to permethrin under laboratory condition [24]. However, evidence of a strong relation with pyrethroid resistance and particularly deltamethrin can be discussed in Latin America [8,23,26]. In addition, no *kdr* phenotype was observed in CAL where the highest proportion of 1011M was observed. We then hypothesized that resistance in CAL is due to metabolic resistance. We then focused on V1016I and F1534C mutations, which are well known to be linked alone or in combination to either deltamethrin or permethrin resistance.

In contrast, GUY and GUA populations have alleles of resistance at the 1016 and 1534 loci. In fact, target site mutations are multiple and likely linked with pyrethroid resistant populations [7,8,30,32] even if they are involved in the interaction with pyrethroids [33]. The effect of each mutation, their synergy or antagonism in *Ae*. *aegypti* resistant phenotypes, has not yet been fully investigated. Recent publications attempted to identify the role of each mutation *in vitro* by using the Xenope oocysts expression model [24]. However, neither S989P, I1011M nor V1016I were related independently to a modification in deltamethrin linkage [24]. F1534C was related to such modification of type I pyrethroid and not to type II. S989P has always been reported in relation with V1016G and may just enhance the effect of this latter mutation. On the other hand, I1011M was related to cypermethrin resistance (type II) in Ceara, Brazil [25] and V1016I was associated to pyrethroid resistance, including in the present work [6,23,27]. This mutation is in fact followed-up as a marker of resistance [34–36]. Even if F1534C was associated with permethrin resistance and not deltamethrin [28,30], Brito et al. [27] demonstrated that double mutants V1016I and F1534C have an enhanced survival rate when exposed to deltamethrin. It is then of primary importance to study mutations at the sodium channel gene scale to identify the real impact of these mutations alone or in combination in pyrethroid resistant phenotypes and eventually model their interaction with the molecules. Metabolic resistance appears to be associated with the activities of CCE, CYP9J and CYP6Z, both CYP450 enzyme families and GSTs [37,38]. CCE and CYP9J enzymes are able to degrade the insecticide whereas CYP6Z such as *CYP6Z8* seems to degrade subsequent products [38]. In the present study, 11 genes from CYP9J and CYP6Z were up-regulated concurrently with eight others from CYP6 which are regularly found in pyrethroid resistant populations, three CCE and two GSTs. Using populations from distinct geographical areas and different levels of deltamethrin resistance revealed the presence of five common genes, including four CYP450s (Fig 2). Whilst close patterns of expression were observed in GUY and GUA populations, they were different from the CAL population. Those results may be associated with the large difference in resistance levels, vector control histories, impact of agricultural practices and different genetic backgrounds. In addition, 21 over-expressed genes found in the present study were also observed in 12 other natural or pyrethroid selected *AE*. *AEGYPTI* populations distributed worldwide and analysed with the “*Aedes* detox” microarray [12,26,39,40] or by others methods (different microarray and quantitative PCR) [41,42] (Table 2). *CYP6BB2*, *CYP6Z6*, *CYP6M11*, *CYP9J23*, *CYP9J9v1*, *CYP9J9v2*, *CYP9J28*, *CYP6CB1*, *CYP9J27*, *CYP9J10*, *GSTE4* were found in six to nine *Ae*. *aegypti* populations in South America and Asia, enhancing their implication in pyrethroid resistance (Table 2). However, only a few functional studies have shown their role in metabolizing pyrethroids [38,41,43]. *CYP6BB2* and *CYP9M6* were identified as degrading permethrin in Kasai et al. [41]. However, none of our studied population exhibited the *CYP9J32* up-regulated gene found in Mexico, Thailand and Vietnam. *CYP9J32* along with *CYP9J24*, *CYP9J26*, and *CYP9J28* are the four enzymes for which the role in degrading permethrin and deltamethrin was demonstrated [43]. CAL population did not show knock-down resistance phenotype (100% knocked-down mosquito after 1h exposure), we then hypothesized the implication of metabolic resistance to explain only 96% mortality after 24h observation. Among nine of the differentially expressed genes in this population, three (*CYP6BB2*, *CYP9M6*, *CYP9J28*) have been proved to degrade pyrethroids. We can assume that those particular genes play a key role in early resistance development of deltamethrin resistance in CAL population. GUY and GUA demonstrate a larger panel of up-regulated detoxifying genes alongside amino-acid changes in the sodium-channel gene, most likely in response to a higher exposure to insecticides, particularly pyrethroids.

**Table 2:** Up-regulated genes found in our study and other pyrethroid resistance populations worldwide. The number and name of populations where they were up-regulated and references are mentioned.

Gene name	Location	References
Cytochrome P450		
*CYP305A6*	French Guiana, Guadeloupe	-
*CYP9J7*	French Guiana, New Caledonia	-
*CYP12F7*	French Guiana, Guadeloupe, New Caledonia, Cuba*	Bariami 2012
*CYP9J6*	French Guiana, Cayman†	Bariami 2012
*CYP6N12*	French Guiana, Cayman†, Puerto-Rico^¥^, Singapore * ^,^ ^¥^	Bariami 2012, Kasai 2014, Reid 2014
*CYP6BB2*	French Guiana, Guadeloupe, New Caledonia, Cuba*, Cayman, *, Puerto-Rico^¥^, Singapore * ^,^ ^¥^	Bariami 2012, Kasai 2014, Reid 2014
*CYP6Z6*	Guadeloupe, Cayman†, Martinique; Puerto-Rico^¥^, Mexico (Calderitas, Lagunitas, Merida), Peru*	Bariami 2012, Marcombe 2009, Saavedra-Rodriguez 2012, Reid 2014
*CYP6M11*	French Guiana, Guadeloupe, Cuba*, Martinique, Puerto-Rico^¥^, Mexico (Merida), Thailand	Bariami 2012, Marcombe 2009, Saavedra-Rodriguez 2012, Strode 2007, Reid 2014
*CYP6N9*	French Guiana, Cuba* †, Puerto-Rico^¥^	Bariami 2012, Reid 2014
*CYP9J31*	French Guiana, Cayman†, Puerto-Rico^¥^	Bariami 2012, Reid 2014
*CYP9J23*	French Guiana, Guadeloupe, Cuba*, Puerto-Rico^¥^ *, Mexico (Merida), Peru	Bariami 2012, Saavedra-Rodriguez 2012, Reid 2014
*CYP9J9v1*	French Guiana, Guadeloupe, Cuba* †, Cayman, Puerto-Rico^¥^, Mexico (Merida)	Bariami 2012, Saavedra-Rodriguez 2012, Reid 2014
*CYP9J9v2*	French Guiana, Guadeloupe, Cuba*, Cayman, Puerto-Rico^¥^ ^,^ Mexico (Merida)	Bariami 2012, Saavedra-Rodriguez 2012, Reid 2014
*CYP9J28*	New Caledonia, Cuba*, Cayman, Puerto-Rico^¥^, Mexico (Merida, Isla Mujeres), Singapore * ^,^ ^¥^	Bariami 2012, Saavedra-Rodriguez 2012, Strode 2007, Kasai 2014, Reid 2014
*CYP6CB1*	French Guiana, Guadeloupe, Cuba* †, Puerto-Rico^¥^ Mexico (Merida), Peru*, Thailand	Bariami 2012, Saavedra-Rodriguez 2012, Strode 2007, Reid 2014
*CYP9J27*	French Guiana, Guadeloupe, New Caledonia, Cayman, Puerto-Rico^¥^, Mexico (Isla Mujeres), Thailand	Bariami 2012, Strode 2007, Reid 2014
*CYP9J10*	French Guiana, Guadeloupe, New Caledonia, Cuba*, Cayman, Mexico (Isla Mujeres), Thailand	Bariami 2012, Strode 2007
*CYP6P12v1*	French Guiana, Mexico (Lagunitas)	Saavedra-Rodriguez 2012
*CYP9M6*	New Caledonia, Thailand, Singapore *,^¥^	Strode 2007, Kasai 2014
Carboxylesterases		
*CCE3*	French Guiana, Guadeloupe, New Caledonia	-
*CCEAE3A*	French Guiana, Guadeloupe, Martinique	Marcombe 2009
*CCEAE3B*	French Guiana, Guadeloupe, Martinique	Marcombe 2009
Glutathione S-transferases		
*GSTE4*	French Guiana, Guadeloupe, Cayman, Mexico (Lagunitas, Merida, Isla Mujeres)	Bariami 2012,Saavedra-Rodriguez 2012, Strode 2007
*GSTE2*	French Guiana, Guadeloupe, Mexico (Isla Mujeres)	Strode 2007

^†^ Genes up-regulated 2 fold in “*Aedes* detox” microarrays;

^¥^ other methods;

*resistant laboratory strain.

Even if detoxification enzymes are considered to be the core of insecticide degradation, other enzymes may play a role in the phenotype of resistance. The serine protease family is related to many processes in insects: digestion [44], interference with penetration and multiplication of the virus in the mosquito (trypsins) [45,46] and also insecticide resistance [47–49]. These enzymes are also studied in the development of novel insecticides In our study, we observed that trypsins are significantly down-regulated. Transcripts AAEL013629-RA, AAEL010196-RA, AAEL010203-RA, AAEL013714-RA, and AAEL010195-RA coding for trypsins, and AAEL013712-RA coding for a Precursor of 5G1 trypsin inhibitor were also down-regulated in the pyrethroid resistant *Ae*. *aegypti* population called Cayman [39] AAEL008079-RA, which was down-regulated in the GUY, GUA and CAL populations, was also down-regulated in the deltamethrin-resistant lab strain CUBA-Delta but up-regulated in the CAYMAN population. Over-expression of two particular trypsins (>4.68-fold vs susceptible) and chymotrypsin (>4.89-fold vs susceptible) in deltamethrin-resistant *Culex pipiens pallens* populations was shown by using microarray and their role in degrading deltamethrin was demonstrated (50). Therefore, opposite results are observed for the same gene family within the *Culicidae* family. Serine proteases are key enzymes in mosquito metabolism and play a role in pyrethroid degradation but their role in the detoxification process must be explored along with its impact on vector competence.

Other enzymes in the detoxification pathway may be of interest in the study of vector competence variability. Indeed, CYP450 enzymes are membrane-bound hemoproteins, which require the activity of NADPH P450 oxidoreductase and sometimes cytochrome b5 to function. Enzyme activity is also regulated by phosphatases, kinases and phosphorylases [50]. In addition, oxido-reduction enzymes are also involved in processing the products of detoxifying enzymes. The presence of up- or down-regulation of those transcripts is then not surprising in our study and are often over transcribed in pyrethroid resistant populations [26,47,51]. Investigating insecticide detoxification pathways is in itself important but the importance is elevated as because some of those enzyme families could mediate arbovirus infection in mosquito cells [52,53]. Particular attention should be paid to these interactions to better understand vector competence and disease dynamics.

We chose to study geographically distinct *Ae*. *aegypti* populations with different levels of resistance to identify which mechanisms may be common and therefore important for resistance development. Amongst the diversity and complexity of resistance, it is essential to identify key markers in order to develop easy-to-use diagnostic tools. Neither the geographic location, resistance levels nor historical use of insecticides can fully explain the distribution of one or another mechanism of resistance. It is then essential to integrate all of the factors that may impact resistance *in vivo* and also *in vitro* to get the best picture, the most reliable data in order to develop predictive tools. In this study, a set of genes were highlighted and which can serve as a basis for further studies on the factors which may induce, develop and maintain the expression of those genes. The role that enzymes may play alone or in association with resistance phenotypes and the consequences of over-expression of those genes on other physiological and cellular processes should then be investigated.

## Supporting Information

S1 Fig Location map of collection sites. (TIF)

S1 Table Quantitative PCR primer list for controlling gene expression in microarray. (DOCX)

S2 Table Allele frequencies within each population and susceptible (S) and resistant (R) group of mosquitoes. Four alleles were found ‘1016V + 1534F’ called S, ‘1016V + 1534C’ (1534 *kdr*) called R1, ‘1016I + 1534 C’ called R2 (1016 *kdr*+1534 *kdr*) and ‘1016I + 1534 F’ called R3 (1016 *kdr*). Number of individual is also mentioned (N).
(DOCX)

S3 Table List of genes significantly up and down regulated in the three studied populations. (XLSX)
