# Supplementary figures and images for: Deltamethrin Resistance Mechanisms in Aedes aegypti Populations from Three French Overseas Territories Worldwide
Source: PLoS Negl Trop Dis. 2015 Nov 20;9(11):e0004226. doi: 10.1371/journal.pntd.0004226 (PMC4654492; doi:10.1371/journal.pntd.0004226)

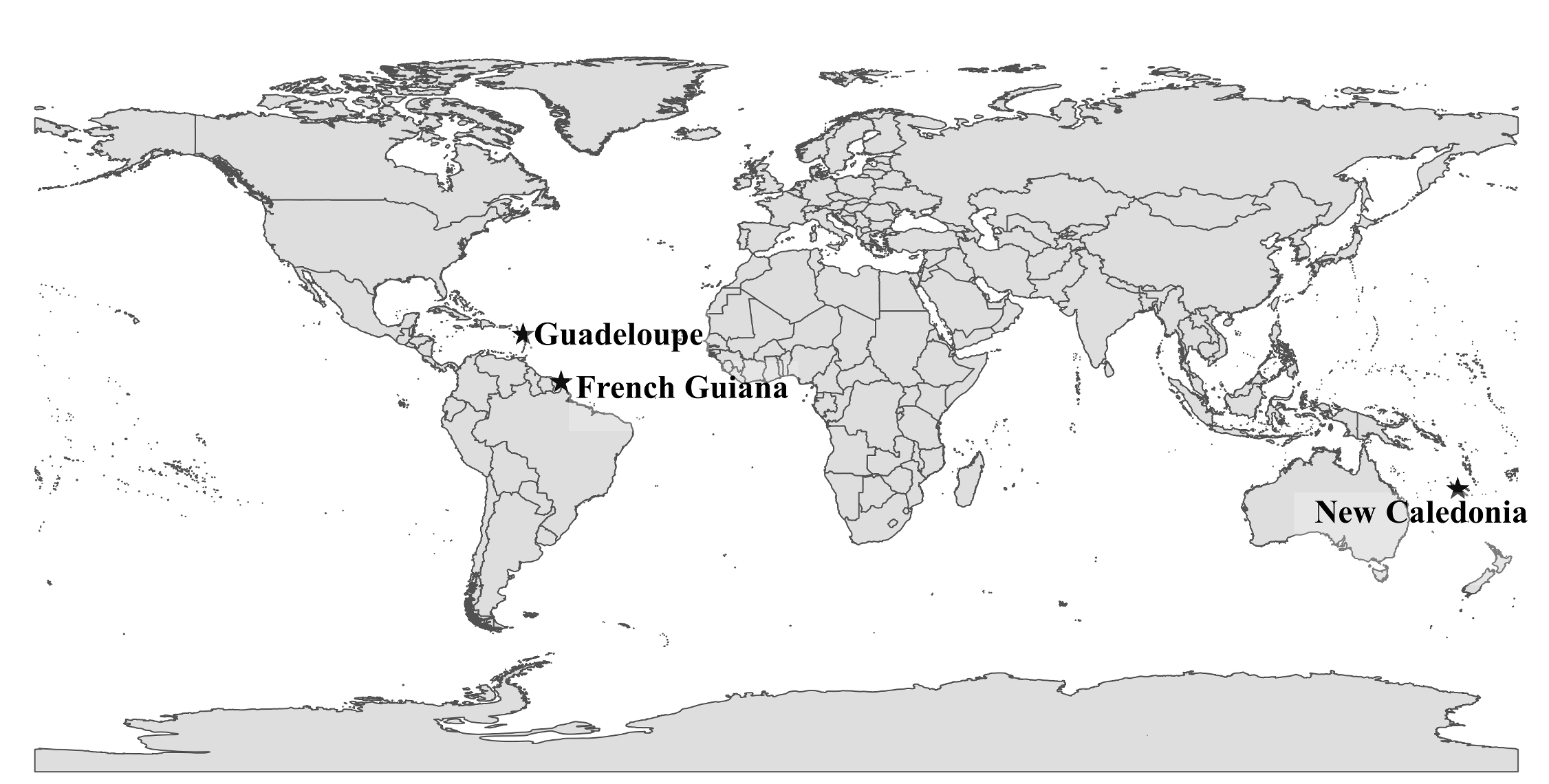

Supplement: S1 Fig — (TIF) [file pntd.0004226.s001.tif]
