# Supplementary material for: Deltamethrin Resistance Mechanisms in Aedes aegypti Populations from Three French Overseas Territories Worldwide
Source: PLoS Negl Trop Dis. 2015 Nov 20;9(11):e0004226. doi: 10.1371/journal.pntd.0004226 (PMC4654492; doi:10.1371/journal.pntd.0004226)
Supplement: S1 Table — (DOCX) [file pntd.0004226.s002.docx]

| **Nom** | **Sequence 5'-3'** |
| --- | --- |
| Cytochrome 014893 For-J | GAGTTGTCAGTGAAACCCTACGG |
| Cytochrome 014893 Rev | GATCGTGGTGCATCGAGTGGATG |
| Cytochrome 006798 For-J | AATACGTACGAGGGATCCAAGA |
| Cytochrome 006798 Rev | CTATCTCCTCCGACCTCGTCCTC |
| Cytochrome 001312 For-J | AGCTTGGCAATGACATCATCAC |
| Cytochrome 001312 Rev | TAAGTCCCTGAAATCCACCAGTG |
| RpS14 For-J | AGGAACTAGCAGAATGGCTCCC |
| RpS14 Rev | ACAGATCCGTGACATGGACGAAG |

S1 Table: quantitative PCR primer list for controlling gene expression in microarray.
