# Supplementary material for: Deltamethrin Resistance Mechanisms in Aedes aegypti Populations from Three French Overseas Territories Worldwide
Source: PLoS Negl Trop Dis. 2015 Nov 20;9(11):e0004226. doi: 10.1371/journal.pntd.0004226 (PMC4654492; doi:10.1371/journal.pntd.0004226)
Supplement: S2 Table — Four alleles were found ‘1016V + 1534F’ called S, ‘1016V + 1534C’ (1534 kdr) called R1, ‘1016I + 1534 C’ called R2 (1016 kdr+1534 kdr) and ‘1016I + 1534 F’ called R3 (1016 kdr). Number of individual is also mentioned (N). (DOCX) [file pntd.0004226.s003.docx]

| **Population** | **Status** | **S** | **R1** | **R2** | **R3** |
| --- | --- | --- | --- | --- | --- |
| **GUY** | S (N=35) | 0.00 | 0.31 | 0.69 | 0.00 |
|  | R (N=15) | 0.00 | 0.10 | 0.90 | 0.00 |
| **GUA** | S (N=33) | 0.21 | 0.16 | 0.63 | 0.00 |
|  | R (N=30) | 0.12 | 0.08 | 0.78 | 0.02 |
| **CAL** | S (N=13) | 1.00 | 0.00 | 0.00 | 0.00 |
|  | R (N=25) | 1.00 | 0.00 | 0.00 | 0.00 |

S2 Table: Allele frequencies within each population and susceptible (S) and resistant (R) group of mosquitoes. Four alleles were found ‘1016V + 1534F’ called S, ‘1016V + 1534C’ (1534 kdr) called R1, ‘1016I + 1534 C’ called R2 (1016 kdr+1534 kdr) and ‘1016I + 1534 F’ called R3 (1016 kdr). Number of individual is also mentioned (N)
